# Disrupted Topological Organization of Functional Networks in Asymptomatic Carotid Plaque Without Significant Carotid Stenosis: A Resting-State fMRI Study

**DOI:** 10.3389/fnhum.2021.685763

**Published:** 2021-08-05

**Authors:** Jia Tuo, Wei He, Shuai Yang, Lihui Liu, Xiaojuan Liu, Hui Liu, Yang Wang, Tao Tang, Jian Xia, Weihua Liao, Yunhai Liu, Qing Huang

**Affiliations:** ^1^Department of Neurology, Chenzhou No.1 People's Hospital, Chenzhou, China; ^2^Hunan Clinical Research Center for Cerebrovascular Disease, Xiangya Hospital, Central South University, Changsha, China; ^3^National Clinical Research Center for Geriatric Disorders, Xiangya Hospital, Central South University, Changsha, China; ^4^Department of Emergency, Xiangya Hospital, Central South University, Changsha, China; ^5^Department of Neurology, Xiangya Hospital, Central South University, Changsha, China; ^6^Department of Radiology, Xiangya Hospital, Central South University, Changsha, China; ^7^Department of Integrated Traditional and Western, Xiangya Hospital, Central South University, Changsha, China

**Keywords:** functional networks, topological organization, carotid plaque, stroke, resting-state fMRI

## Abstract

**Purpose:** Previous studies have found that there are significant changes in functional network properties for patients with moderate to severe carotid artery stenosis. Our study aimed to explore the topology properties of brain functional network in asymptomatic patients with carotid plaque without significant stenosis.

**Methods:** A total of 61 asymptomatic patients with carotid plaque (mean age 61.79 ± 7.35 years) and 25 healthy control subjects (HC; 58.12 ± 6.79 years) were recruited. General data collection, carotid ultrasound examination and resting state functional magnetic resonance imaging were performed on all subjects. Graph-theory was applied to examine the differences in the brain functional network topological properties between two groups.

**Results:** In the plaque group, E_loc_(*P* = 0.03), γ (*P* = 0.01), and σ (*P* = 0.01) were significantly higher than in the HC group. The degree centrality of left middle frontal gyrus and the nodal efficiency of left middle frontal gyrus and right inferior parietal angular gyrus were significantly higher in the plaque group than in HC. The degree centrality and betweenness centrality of right middle temporal gyrus, as well as the nodal efficiency of right middle temporal gyrus, were significantly lower in the plaque group than in HC.

**Conclusions:** The brain functional networks of patients with carotid plaques differ from those of healthy controls. Asymptomatic patients with carotid plaques exhibit increased local and global connectivity, which may reflect subtle reorganizations in response to early brain damage.

## Introduction

Stroke is a major cause of death and disability worldwide (Johnston et al., 2009) with ischemic stroke (IS) representing ~70% of cases (Tsai et al., 2013; Sun et al., 2014). Studies have shown that 10–20% of IS occurs secondary to carotid atherosclerosis. With the continuous improvement of neuroimaging technologies, more and more researchers are studying the changes to brain networks that occur with neurological diseases, such as Parkinson's disease and stroke. Several studies have shown that many risk factors of IS are significantly correlated with brain functional network changes (Lv et al., 2016; Yuan et al., 2016; Bi et al., 2017; Naumczyk et al., 2017).

Previous imaging studies have investigated the effects of carotid stenosis on the brain, focusing on structural damage and functional connectivity (Porcu et al., 2020). Asymptomatic carotid stenosis has been shown to have a detrimental effect on neuronal activity and regional functional connections (Rocque et al., 2012). Other studies have reported changes in connectivity and topological properties of brain functional networks in patients with carotid stenosis (Cheng et al., 2012; Chang et al., 2016; Wang and Xiao, 2017). In addition, there is evidence that patients with asymptomatic carotid artery stenosis display cognitive impairment (Mathiesen et al., 2004; Romero et al., 2009) and that carotid artery stenosis is an independent risk factor for cognitive decline (Buratti et al., 2014; Wang et al., 2016a). Many studies have demonstrated a relationship among intima-media thickness (IMT), carotid plaques, and cognitive impairment (Rossetti et al., 2015; Wang et al., 2016b).

Graph theory is a powerful method for quantifying the organization of brain connectivity. It regards brain networks as graphs composed of nodes and edges. The brain regions (voxels) are considered as nodes, and structural or functional connectivity among the nodes is regarded as edges. The concept of “small-world” networks was first introduced by Watts and Strogatz (1998). A small-world network combines the advantages of regular and random networks to yield one that has faster information processing speed than a regular network, and higher information processing efficiency than a random network. It has been reported that brain networks have small-world properties (Achard et al., 2006; He et al., 2007).

Carotid atherosclerosis proceeds in stages, with carotid plaque preceding carotid stenosis. Many studies have explored brain changes in patients with severe carotid stenosis. Considering that brain alterations are long, slow processes, we suspect that changes to the brain networks are already present in patients with carotid plaque. The objective of this study is to explore the changes in the brain functional network of patients, who have a normal cognitive function, with asymptomatic carotid plaques.

## Materials and Methods

### Participants

All subjects in this study were recruited from participants of the Stroke Screening and Prevention Project conducted at Xiangya Hospital (registered between December 2015 and June 2018), a comprehensive project assessing the risk of stroke in a population aged over 40 years old. Subjects were interviewed by researchers to gather demographic information including age, gender, education level, alcohol consumption, smoking history, past medical history, and medications. A total of 2,015 subjects were enrolled in the project, of whom 1,154 underwent carotid ultrasound tests. After screening, 132 subjects were willing to undergo brain MRI. The inclusion criteria were as follows: (1) aged from 40 to 70 years old; (2) right-handed; (3) Mini-Mental State Examination (MMSE) scores ≥27 and Montreal cognitive assessment (MoCA) scores ≥26; (4) no previous diagnosis of transient IS; (5) no organic or mental illness; and (6) no drug dependence. Participants were excluded if they had any MRI contraindications, had a history of neurological or mental diseases or substance abuse, or were currently pregnant. The degree of carotid artery stenosis of subjects in the plaque group (PA) was <50%. Subjects in the healthy control group (HC) were required to have no carotid plaque. All subjects signed the informed consent. All procedures were approved by the ethics committee of Xiangya School of Medicine, Central South University.

### Blood Analysis

Venous blood was collected from the antecubital vein in the morning before breakfast, following an overnight fast of 12–14 h. All blood samples were subjected to routine laboratory tests including assays for blood sugar, serum total cholesterol, serum triglycerides, serum high-density lipoprotein (HDL) cholesterol, serum low-density lipoprotein (LDL) cholesterol, and homocysteine (Hcy).

### Carotid Ultrasound Acquisition

Examination of carotid plaques was performed by the same experienced radiologist using an ultrasound scanner (5-MHz Linear Array transducer; iU22, Philips Ultrasound, Bothell, WA) in both the right and left common carotid arteries (CCAs) and bifurcations. The distance from the media-adventitia interface to the intima-lumen interface was defined as the IMT (Crouse et al., 1995). Radiologists examined each carotid wall and segments from continuous angles to obtain the thickest intima-media site. A PA was defined as a focal structure with an IMT > 1.5 mm. The carotid IMT (CIMT) has shown to be an effective index to reflect atherosclerosis (Crouse et al., 1995) and was defined, in this study, as the average of bilateral CCA, carotid bulb, and bifurcations IMTs in a region free of PA. Ultrasonographic findings of plaques can be found in Supplementary Table 1.

### MRI Data Acquisition

MRI data were acquired on a GE 3.0 MRI scanner (General Electric systems, USA) with a 12-channel head coil. The MRI scanning included T1-weighted imaging (T1WI), T2-weighted imaging (T2WI), and fluid-attenuated inversion recovery (FLAIR) sequences. Resting-state (rs) fMRI data were obtained using a T2-weighted gradient-echo, echo-planar imaging (EPI) sequence with the following parameters: repetition time (TR) = 2,000 ms, echo time (TE) = 30 ms, flip angle = 90°, slice thickness = 4 mm, acquisition matrix = 64 × 64, voxel size = 3 × 3 × 3 mm^3^. For each subject, 32 continuous axial slices per volume and a total of 360 volumes were acquired in 6 min. During the scanning, all lights were switched off and participants placed cotton in their ears to reduce sound. Subjects were instructed to relax in a supine position, breathing normally, and keeping still while not thinking about anything or falling asleep. Schelten's scale was used to evaluate the degree of white matter lesions.

### MRI Data Preprocessing

The preprocessing of rs-fMRI data was conducted using the GRETNA toolbox (http://www.nitrc.org/projects/gretna/) implemented in MATLAB (Mathworks, Natick, MA). The specific steps were as follows: (1) slice timing correction: removing the first five volumes, (2) realignment for head motion correction, (3) spatial normalization to the Montreal Neurological Institute template, (4) linear temporal detrending, (5) regress out covariates (global signals, mean white matter signals, cerebrospinal fluid signals, and 24 head-motion realignment parameters time series), (6) temporal band-pass filter (0.01–0.08 Hz), and (7) scrubbing (participants with a displacement >3 mm or an angular rotation >3 degrees in any direction of MRI data were excluded).

### Functional Network Analysis

The pre-processed rs-fMRI image of the whole brain of each subject was divided into 90 regions of interest (ROI) by using the automated anatomical labeling (AAL) atlas. Each region was regarded as a node, and the time-series data for each voxel within a region were averaged to yield a mean signal intensity. The Pearson's correlation coefficient (r) between each pair of the ROIs was calculated, and a 90 × 90 correlation matrix was constructed. Each network metric extracted across the specified sparsity range is represented by a curve that depicts the changes in network metrics as a function of network density (threshold).

The topological properties of a functional network are divided into global and node-based parameters. We analyzed five global and six node-based parameters. Global parameters comprised global efficiency (E_glob_), local efficiency (E_loc_), clustering coefficient (Cp), characteristic path length (Lp), and small-worldness (s), which itself comprised gamma (γ), lambda (λ), and sigma (σ). Node-based parameters included degree centrality, betweenness centrality, clustering coefficient, efficiency, E_loc_, and shortest Lp. Brief descriptions of each of these parameters can be found in the Data Supplement, and mathematical definitions can be found in a previously published study (Rubinov and Sporns, 2010).

### Statistical Analysis

Continuous variables with normal distributions were expressed as mean (SD), continuous variables without normal distributions were expressed as median (interquartile range), and categorical variables were expressed as percentages. Two-sample *t*-tests were performed to evaluate differences in demographic characteristics and network parameters between the two groups. Chi-square tests were used to analyze differences between categorical variables, and the Whitney *U*-test was used to explore differences between measurements, which did not conform to a normal distribution. These analyses were performed using SPSS 22.0 and GRETNA software. The *p* < 0.05 was considered statistically significant. The FDR-corrected thresholds were set at a *p* < 0.05.

## Results

### Demographic and Clinical Characteristics

A total of 132 subjects were recruited for the study. About 20 subjects were excluded because of PAs in the subclavian artery, 18 because of MRI displacement and angular rotation, and 8 because of incomplete MRI data. The final study population consisted of 61 subjects with PAs and 25 HCs. The demographic and clinical characteristics of the study population are summarized in Table 1. Two-sample *t*-tests showed that there were significant differences between the two groups in terms of CIMT (*P* < 0.001). No significant differences were found for age, gender, years of education, BMI, or traditional risk factors of atherosclerosis, such as systolic blood pressure, LDL-C, HDL-C, triglycerides, fasting glucose, and Hcy.

**Table 1:** Demographic and clinical characteristics of the participants.

**Characteristic**	**Plaque group**	**Control group**	**T/χ^**2**^/*Z* value**	**df**	***p*-value**	**Cohen's *d***
Age, mean (SD) (y)	61.79 (7.35)	58.12 (6.79)	1.56^*^	84	0.12^*^	–
Male, No./total No. (%)	24/61 (39.34)	12/25 (48.00)	0.55^#^	1	0.46^#^	–
BMI, mean (SD) (kg/m^2^)	24.97 (3.93)	24.76 (4.25)	0.22^*^	84	0.83^*^	–
Education, mean (SD), y	9.13 (2.51)	9.52 (2.33)	−0.67^*^	84	0.51^*^	–
Hypertension, No./total No (%)	24/61 (39.34)	11/25 (44.00)	0.16^#^	1	0.69^#^	–
Smoking, No./total No (%)	20/61 (32.79)	10/25 (40.00)	0.406^#^	1	0.52^#^	–
SBP, mean (SD) (mm Hg)	136.97 (18.35)	133.96 (15.72)	0.72^*^	84	0.48^*^	–
DBP, mean (SD) (mm Hg)	85.93 (9.70)	84.32 (11.88)	0.66^*^	84	0.51^*^	–
Fasting glucose, mean (SD) (mmol/L)	6.01 (1.17)	5.69 (0.77)	1.55^*^	84	0.14^*^	–
Total cholesterol, mean (SD) (mmol/L)	5.39 (1.21)	5.45 (0.87)	−0.24^*^	84	0.81^*^	–
Triglyceride, mean (SD) (mmol/L)	2.03 (1.36)	2.06 (0.91)	−0.11^*^	84	0.91^*^	–
HDL-C, mean (SD) (mmol/L)	1.45 (0.49)	1.45 (0.43)	0.04^*^	84	0.99^*^	–
LDL-C, mean (SD) (mmol/L)	3.24 (0.96)	3.23 (0.69)	0.05^*^	84	0.96^*^	–
Hcy, mean (SD) (μmol/L)	12.96 (6.19)	12.66 (3.70)	0.22^*^	84	0.83^*^	–
CIMT, mean (SD) (cm)	0.81 (0.10)	0.70 (0.06)	0.60^*^	84	<0.001^*^	1.33
MMSE, (P75, P25), (S)	28 (27, 29)	28 (28, 30)	−1.43^‡^	–	0.15^‡^	–
MoCA, (P75, P25), (S)	27 (26, 28)	27 (27, 28)	−0.93^‡^	–	0.35^‡^	–
HAMD, (P75, P25), (S)	3 (2, 6)	2 (2.5, 5)	−0.40^‡^	–	0.67^‡^	–
HAMA, (P75, P25), (S)	3 (2.5, 5)	2 (1.5, 5)	−0.68^‡^	–	0.43^‡^	–
Scheltens, (P75, P25), (S)	2 (1, 4)	2 (0, 4)	−0.34^‡^	–	0.73^‡^	–

*BMI, body mass index; SBP, systolic blood pressure; DBP, diastolic blood pressure; HDL, high-density lipoprotein; LDL, low-density lipoprotein; Hcy, homocysteine; CIMT, carotid intima-media thickness; MMSE: mini-mental state examination; MoCA, Montreal Cognitive Assessment; HAMD, Hamilton Depression Scale; and HAMA, Hamilton Anxiety Scale*;

* *P-value and T-value were obtained by two-sample t-test*;

# *p-value and*

*χ^2^ value were obtained by χ^2^ test*;

‡ *p-value and Z-value were obtained by Whitney U-test; p < 0.05, respectively*.

### Global Parameters

**Figure 1:**
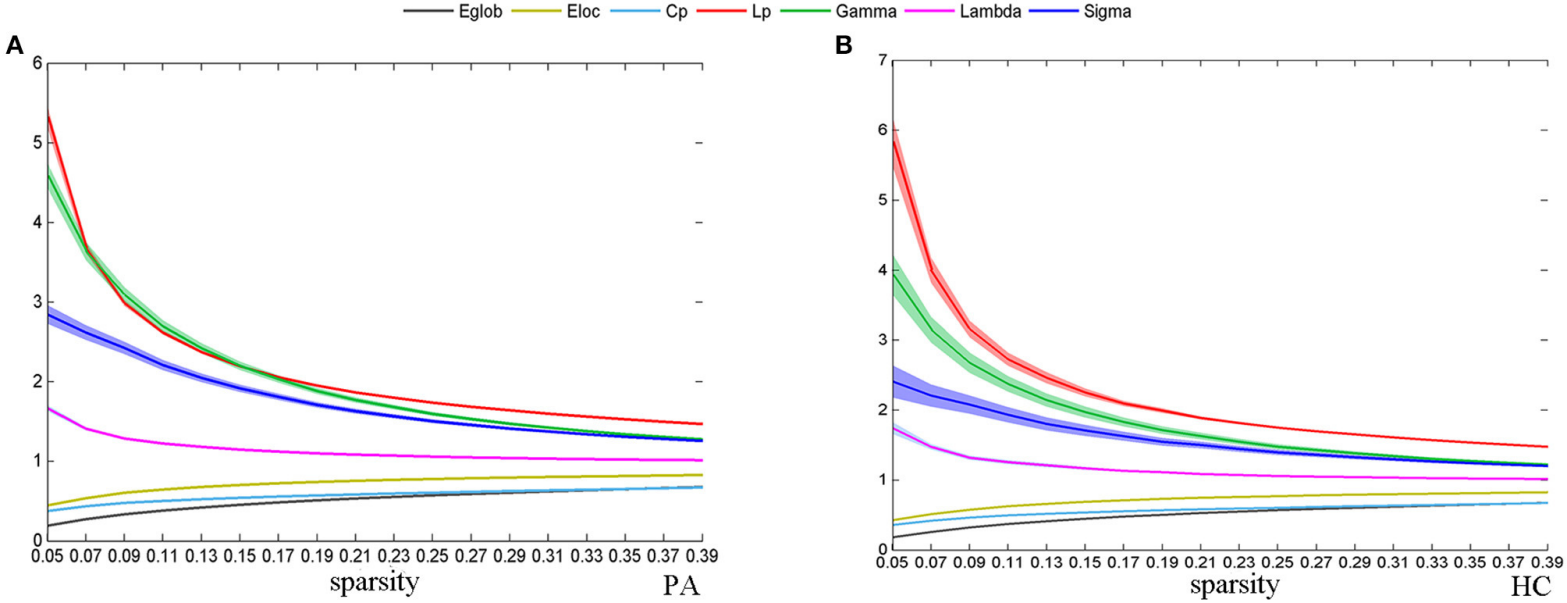
The change of global network topological properties with the change of sparsity. The solid line represents the averaged values across subjects, and the light-colored area indicates the range of one SD. Plots show the relationship between the r threshold and global network topological properties for the plaque group (PA) **(A)** and healthy control group (HC) **(B)**.

Across the specified density range (0.05:0.02:0.4), the functional network of both groups followed a small-world organization; that is, λ was close to 1, γ was >1, yielding a σ > 1. As sparsity increased, E_glob_, E_loc_, and Cp increased and Lp, γ, and σ decreased (Figure 1).

**Figure 2:**
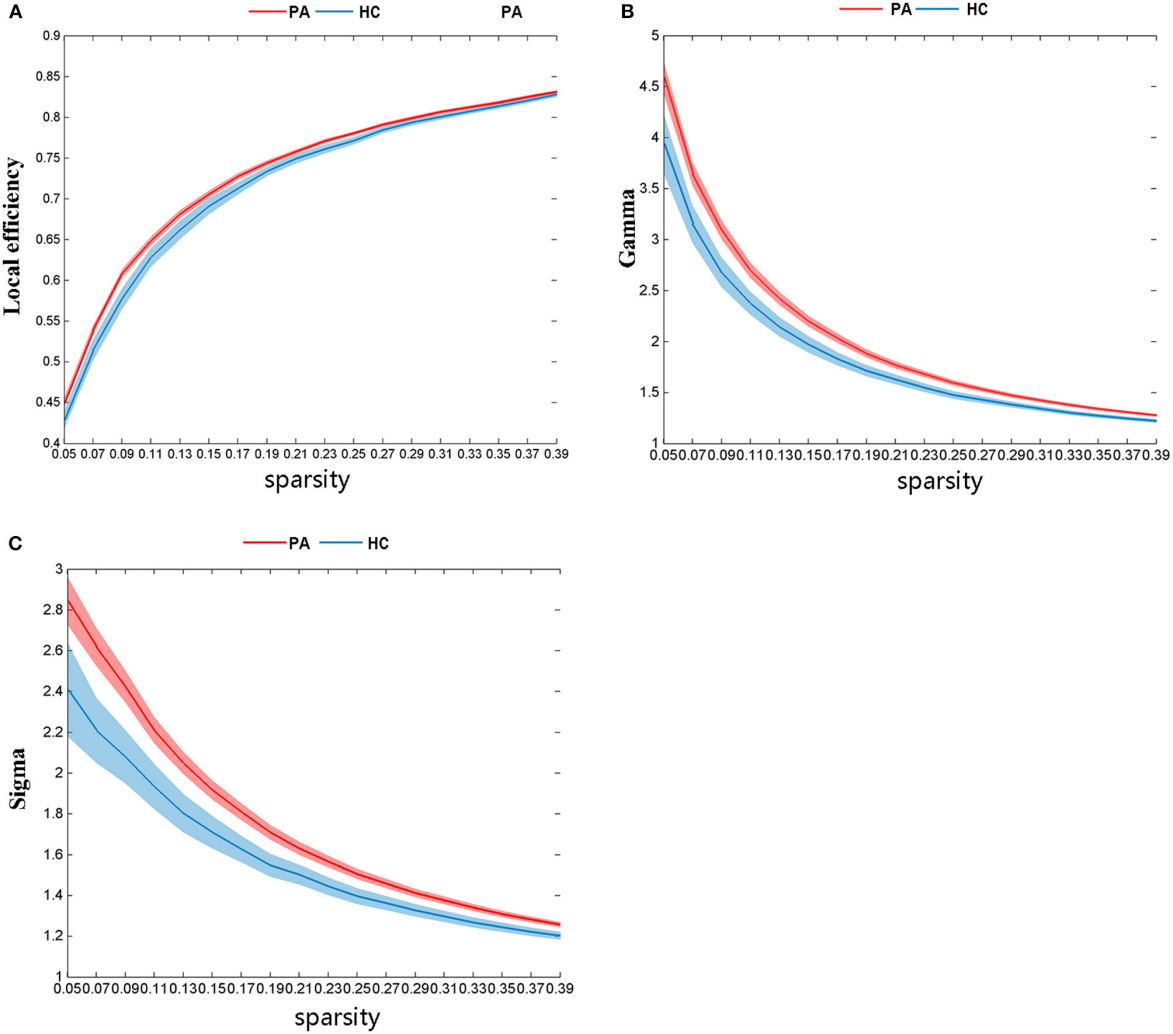
There are significant differences in global network topological properties between the PA group and HC group. The HC group is represented by the blue line and the PA group by the red. **(A)** local efficiency (E_loc_), **(B)** gamma (γ), and **(C)** sigma (σ).

There was significant group differences in global topological properties, including E_loc_, γ, and σ. The E_loc_ (*P* = 0.03), γ (*P* = 0.01), and σ (*P* = 0.01) values of the PA group were higher than those of the HC group (Figure 2); however, differences between the PA group and the HC group for E_glob_ (*P* = 0.13), Cp (*P* = 0.53), Lp (*P* = 0.06), and λ (*P* = 0.06) were not significant.

### Nodal Parameters

**Figure 3:**
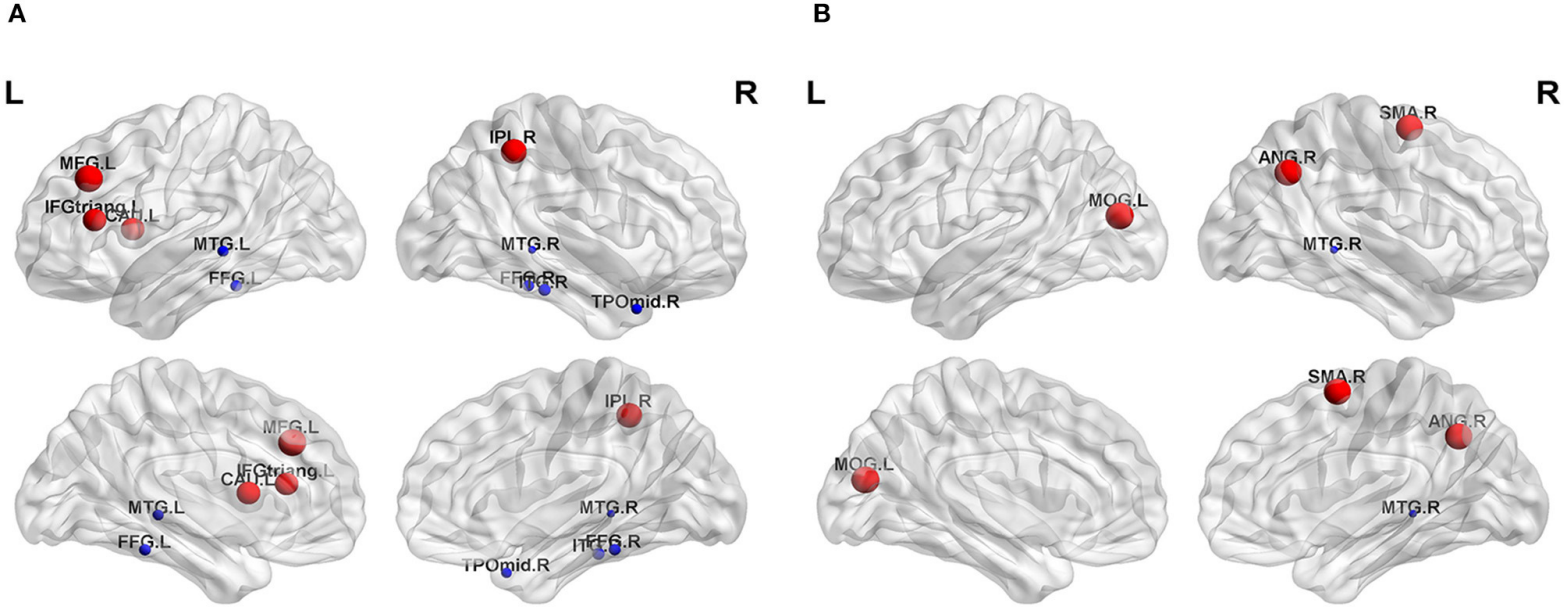
Significant differences in nodal topological properties, degree centrality **(A)** and betweenness centrality **(B)**, were observed between the PA group and HC group. Red areas represent nodes for which PA > HC, blue for which PA < HC (*P* < 0.05, FDR-uncorrected). L, left; R, right; MFG, middle frontal gyrus; IFGtriang, inferior frontal gyrus triangular part; IPL, inferior parietal, but supramarginal and angular gyri; CAU, caudate nucleus; FFG, fusiform gyrus; MTG, middle temporal gyrus; TPOmid, temporal pole: middle temporal gyrus; ITG, inferior temporal gyrus; SMA, supplementary motor area; MOG, middle occipital gyrus; ANG, angular gyrus.

**Figure 4:**
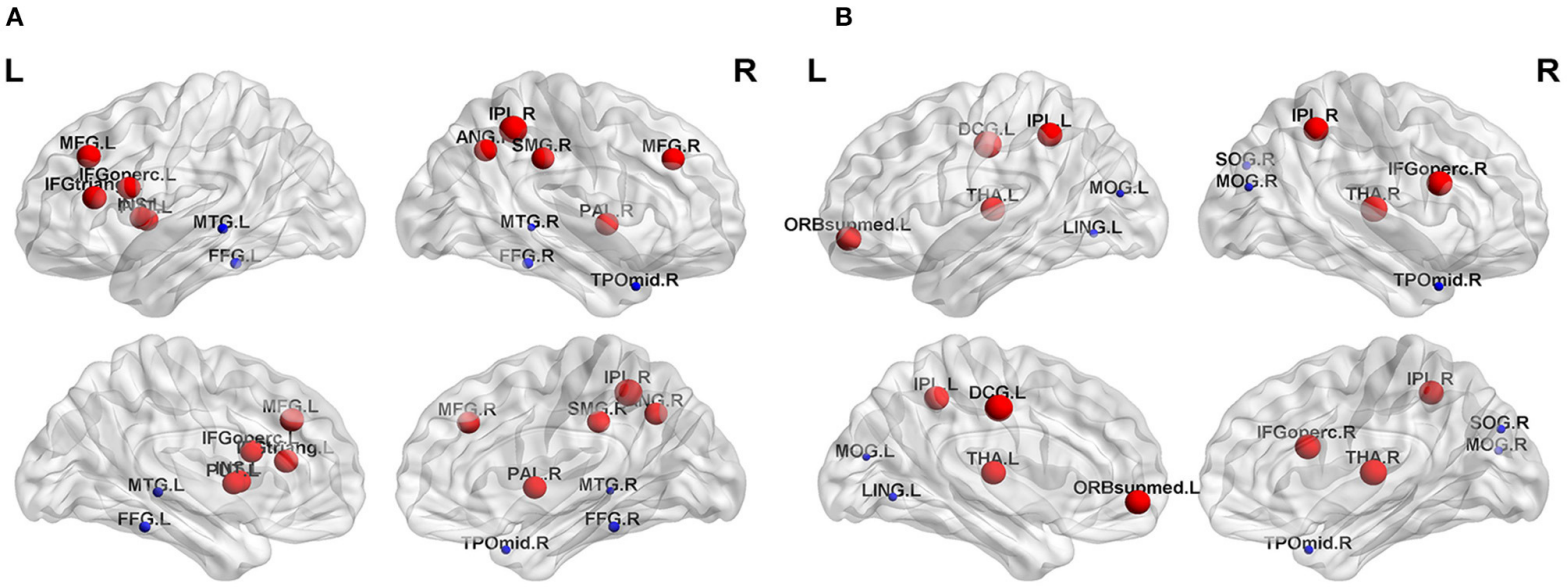
Significant differences in nodal topological properties, efficiency **(A)** and local efficiency **(B)**, were observed between the PA group and HC group. Red areas represent nodes for which PA > HC, blue for which PA < HC (*P* < 0.05, FDR-uncorrected). L, left; R, right; MFG, middle frontal gyrus; IFGoperc, inferior frontal gyrus, opercular part; INS, insula; IPL, inferior parietal, but supramarginal and angular gyri; SMG, supramarginal gyrus; ANG, angular gyrus; PUT, lenticular nucleus, putamen; PAL, lenticular nucleus, pallidum; FFG, fusiform gyrus; MTG, middle temporal gyrus; TPOmid, temporal pole: middle temporal gyrus; IFGoperc, inferior frontal gyrus, opercular part; ORBsupmed, superior frontal gyrus, medial orbital; DCG, median cingulate and paracingulate gyri; THA, thalamus; LING, lingual gyrus; SOG, superior occipital gyrus; MOG, middle occipital gyrus.

The nodal topological parameters in 90 brain regions were compared across groups, and the changes observed for nodal parameters in the PA group were bidirectional (*P* < 0.05, FDR corrected). The degree centrality of the PA group was significantly higher in the left middle frontal gyrus (MFG.L), but lower in the right middle temporal gyrus (MTG.R) (Table 2, Figure 3). Betweenness centrality in MTG.R of the PA group was significantly lower than that of the HC group (Table 2, Figure 3). In the PA group, nodal efficiency in MFG.L and right inferior parietal angular gyrus (IPL.R) was higher than in HCs, and in the MTG.Rs was lower (*P* < 0.05) (Table 3, Figure 4). There was no significant difference between groups in E_loc_, clustering coefficient, or shortest Lp (Table 4) (*P* > 0.05).

**Table 2:** Brain regions with differences in degree and betweenness centrality between the plaque group (PA) and the healthy control group (HC).

**Degree**						**Betweenness**					
**PA > HC**	**PA < HC**	**T**	**df**	***p*-value**	**Cohen's *d***	**PA > HC**	**PA < HC**	**T**	**df**	***p*-value**	**Cohen's *d***
MFG.L		>3.410	84	<0.001^*^	>0.744	SMA.R		2.083	84	0.040	–
IFGtriang. L		2.121	84	0.037	–	MOG.L		2.370	84	0.020	–
IPL.R		2.952	84	0.004	–	ANG.R		2.143	84	0.035	–
CAU.L		2.168	84	0.033	–		MTG.R	>3.410	84	<0.001^*^	>0.744
	FFG.L	2.510	84	0.014	–						
	FFG.R	2.510	84	0.014	–						
	MTG.L	2.720	84	0.008	–						
	MTG.R	>3.410	84	<0.001^*^	>0.744						
	TPOmid. R	2.880	84	0.005	–						
	ITG.R	2.287	84	0.025	–						

*L, left; R, right; MFG, middle frontal gyrus; IFGtriang, inferior frontal gyrus, triangular part; IPL, inferior parietal, but supramarginal and angular gyri; CAU, caudate nucleus; FFG, fusiform gyrus; MTG, middle temporal gyrus; TPOmid, temporal pole: middle temporal gyrus; ITG, inferior temporal gyrus; SMA, supplementary motor area; MOG, middle occipital gyrus; and ANG, angular gyrus*;

* *p < 0.05, FDR-corrected*.

**Table 3:** Brain regions with differences in efficiency and E_loc_ between the PA group and HC.

**Efficiency**						**Local efficiency**					
**PA > HC**	**PA < HC**	**T**	**df**	***p*-value**	**Cohen's *d***	**PA > HC**	**PA < HC**	**T**	**df**	***p*-value**	**Cohen's *d***
MFG.L		>3.410	84	<0.001^*^	>0.744	IFGoperc.R		2.287	84	0.025	–
MFG.R		2.035	84	0.045	–	ORBsupmed.L		2.155	84	0.034	–
IFGoperc.L		2.168	84	0.033	–	DCG.L		2.880	84	0.005	–
IFGtriang.L		2.483	84	0.015	–	IPL.L		2.035	84	0.045	–
INS.L		2.143	84	0.035	–	IPL.R		2.050	84	0.043	–
IPL.R		>3.410	84	<0.001^*^	>0.744	THA.L		1.988	84	0.050	–
SMG.R		2.013	84	0.047	–	THA.R		2.555	84	0.012	–
ANG.R		2.065	84	0.042	–		LING.L	2.266	84	0.026	–
PUT.L		1.988	84	0.050	–		TPOmid.R	2.131	84	0.036	–
PAL.R		2.210	84	0.030	–		SOG.R	2.240	84	0.028	–
	FFG.L	2.121	84	0.037	–		MOG.L	2.510	84	0.014	–
	FFG.R	2.240	84	0.028	–		MOG.R	2.316	84	0.023	–
	MTG.L	2.483	84	0.015	–						
	MTG.R	3.410	84	0.001^*^	0.744						
	TPOmid.R	2.810	84	0.006	–						

*L, left; R, right; MFG, middle frontal gyrus; IFGoperc, inferior frontal gyrus, opercular part; INS, insula; IPL, inferior parietal, but supramarginal and angular gyri; SMG, supramarginal gyrus; ANG, angular gyrus; PUT, lenticular nucleus, putamen; PAL, lenticular nucleus, pallidum; FFG, fusiform gyrus; MTG, middle temporal gyrus; TPOmid, temporal pole: middle temporal gyrus; IFGoperc, inferior frontal gyrus, opercular part; ORBsupmed, superior frontal gyrus, medial orbital; DCG, median cingulate and paracingulate gyri; THA, thalamus; LING, lingual gyrus; SOG, superior occipital gyrus; and MOG, middle occipital gyrus*;

* *p < 0.05, FDR-corrected*.

**Table 4:** Brain regions with differences in clustering coefficient and shortest path length (Lp) between the PA group and HC.

**Clustering coefficient**						**Shortest path length**					
**PA > HC**	**PA < HC**	**T**	**df**	***p*-value**	**Cohen's *d***	**PA > HC**	**PA < HC**	**T**	**df**	***p*-value**	**Cohen's *d***
THA.L		2.168	84	0.033	–		DCG.L	3.410	84	0.001	0.744
THA.R		2.810	84	0.006	–						
	LING.L	2.266	84	0.026	–						
	SOG.R	2.410	84	0.018	–						
	MOG.L	2.392	84	0.019	–						
	MOG.R	2.510	84	0.014	–						
	IOG.L	2.097	84	0.039	–						

*L, left; R, right; THA, thalamus; LING, lingual gyrus; SOG, superior occipital gyrus; MOG, middle occipital gyrus; IOG, inferior occipital gyrus; and DCG, median cingulate and paracingulate gyri*.

## Discussion

In the present study, participants with the carotid plaque and HCs all displayed functional networks that followed a small-world organization across a range of densities. This observation is in alignment with previous findings (Salvador et al., 2005; Bassett et al., 2008). Small-world networks can maintain the optimal balance of separation and integration of information with low energy and wiring costs (Liao et al., 2017) and is well-suited for complex brain dynamics. The present results also revealed significant differences in global topological properties of functional networks between the two groups after controlling for gender, age, and education level. Previous studies have shown that many risk factors of cerebrovascular disease can alter brain function (Musen et al., 2012; Haight et al., 2015; Lande et al., 2017; Cheng and Rolls, 2019). In this study, we found that carotid plaque, a major risk factor of cerebrovascular disease, affected the topological properties of the brain functional network.

The local efficiency and γ values are indicators that reflect the efficiency of local information processing in the network. The σ value indicates the strength of s. E-loc, γ, and σ values were significantly higher in the PA group than in the HC group. These changes in s suggest that the functional network of the PA group may be more efficient at processing, integrating, and transmitting the information. This is in line with previous hypotheses that such changes might reflect a compensatory mechanism by the PA group to maintain normal function in the early stages of cognitive decline (van Duinkerken et al., 2012). These potential changes of small-world properties in the PA group break the balance of the original brain network, and their changes may only be to maintain the normal level of information transfer and communication.

Carotid plaque and carotid stenosis reflect different stages of carotid atherosclerosis. A recent review of the effects of carotid stenosis on the brain and cognition (Porcu et al., 2020) summarized the disrupted functional connectivity and cognitive impairment in patients with carotid stenosis (Wang and Xiao, 2017; Gao et al., 2019). Many studies have reported that the cerebrovascular system activates a series of compensatory mechanisms to overcome the cerebral hypoperfusion that is induced by carotid stenosis (Shakur et al., 2014; Zarrinkoob et al., 2019). This often manifests as rearrangement of the cerebral circulation and brain function in patients with carotid artery stenosis. Given that no participants in this study had significant stenosis (more than 50%), these results suggest that functional networks were reorganized even before the onset of serious stenosis.

Many studies illustrated that both carotid stenosis and carotid plaque had a strong relationship with cognitive decline. In the Framingham Offspring study, patients with carotid stenosis had poorer performance on executive function (Romero et al., 2009). Compared with the mild to moderate carotid stenosis group, patients with severe carotid stenosis had a lower MMSE score (Martinić-Popović et al., 2009). Furthermore, carotid endarterectomy and carotid artery stent implantation can even change the cognitive impairment of patients with carotid stenosis (Fan et al., 2014; Heyer et al., 2014). We studied a group of patients with carotid plaques whose cognitive function was normal, and these results showed that there were already changes in brain function. These findings suggest that changes to brain function may emerge before subjective symptom onset in patients with carotid plaques. Early detection of the brain functional changes could be clinically beneficial for patients with carotid plaques since early intervention greatly improves prognosis. rs-fMRI is an effective method to detect early brain changes in patients with atherosclerosis. In addition to offering a potential diagnostic aid, the present study also provides a theoretical framework for the impairments in memory, executive function, and attention in patients with carotid atherosclerosis, that is, the disruption of local functional networks. Follow-up experiments will further explore the imaging markers of early large artery atherosclerotic cerebral infarction and accompanying vascular cognitive impairment.

The present study found that there were substantial differences in the nodal parameters of functional networks in several regions, including frontal, temporal, and occipital lobes. This is not completely consistent with the previous study. One study suggested that the increase of IMT was significantly related to the decreased function of the middle frontal gyrus, and they believed that this brain dysfunction might be the early change of vascular dementia in patients with carotid atherosclerosis (Haight et al., 2015). The results of this study are consistent with the findings of another study that there was increased functional connectivity in some brain regions in patients with subcortical vascular mild cognitive impairment without dementia (Muller et al., 2012). In addition, the decrease of node function in plaque patients was mainly located in the middle temporal gyrus, inferior temporal gyrus, and middle occipital gyrus. Indeed, whether the function decreases or increases, it breaks the local balance of the original network. The change of attributes in local regions may correspond to changes in relevant brain functions. These regions are thought to be involved in the cognitive control network, including memory function, emotion management, and executive function, and, indeed, there may have been differences in memory function, emotion management, and executive function between the two groups. Previous studies have shown that carotid atherosclerosis is associated with memory function (Guo et al., 2009; Suemoto et al., 2015; Naumczyk et al., 2017). In future studies, we hope to further explore this relationship, perhaps by correlating functional network changes with measures of cognitive control.

There were several limitations to this study. First, the study was a cross-sectional observational study with a small sample size for the HC group. Thus, the ability to identify causal relationships between functional network changes and plaques was limited. A longitudinal cohort study with a larger sample size is necessary to validate and build upon these findings. Second, the associations between network properties and clinical data were not assessed. Third, no thorough assessments of IQ or cognition were conducted, and the intelligence of participants was merely estimated by MMSE and MoCA scores. Finally, all subjects in this study were over 40 years of age, so it will be necessary to expand the research target population and explore the influence of age and other potential confounding factors.

## Conclusion

Graph theory analysis showed that the brain functional network in patients with the carotid plaque as well as HCs both displayed small-world attributes. Global topological properties of the functional network and local topological properties of the frontotemporal area were significantly different between the two groups. fMRI and graph theory offer potential as diagnostic aids for detecting early brain changes in patients with carotid atherosclerosis.

## Data Availability Statement

The raw data supporting the conclusions of this article will be made available by the authors, without undue reservation.

## Author Contributions

QH and YL: guarantor of integrity of the entire study. QH: study concepts, study design, definition of intellectual content, data analysis, statistical analysis, and manuscript preparation. JT: data analysis, statistical analysis, and manuscript preparation. WH, SY, LL, XL, and HL: data acquisition. YW, TT, JX, and WL: data analysis. All authors contributed to the article and approved the submitted version.

## Conflict of Interest

The authors declare that the research was conducted in the absence of any commercial or financial relationships that could be construed as a potential conflict of interest.

## Publisher's Note

All claims expressed in this article are solely those of the authors and do not necessarily represent those of their affiliated organizations, or those of the publisher, the editors and the reviewers. Any product that may be evaluated in this article, or claim that may be made by its manufacturer, is not guaranteed or endorsed by the publisher.
