# Supplementary material for: Disrupted Topological Organization of Functional Networks in Asymptomatic Carotid Plaque Without Significant Carotid Stenosis: A Resting-State fMRI Study
Source: Front Hum Neurosci. 2021 Aug 5;15:685763. doi: 10.3389/fnhum.2021.685763 (PMC8375554; doi:10.3389/fnhum.2021.685763)
Supplement: Supplementary file 1 [file Data_Sheet_1.docx]

**Supplementary Data**

The definitions of network parameters:

For a given graph N composed of n nodes, the **Degree** of a node “i” is defined as:

$$k_{i}=\sum_{j\in N} a_{ij}$$

where a_ij_ is the connection status between node i and j: a_ij_ = 1 if link exist, otherwise a_ij_ = 0.

The **Clustering coefficient** represents how many nearest neighbors of node i are connected to each other as well, and is defined as [^1^](#_ENREF_1):

$$C_{i}=\sum_{i\in N} \frac{2t_{i}}{k_{i}(k_{i}-1)means}$$

where t_i_ is the number of triangles around a node i.

The **Local efficiency** represents the efficiency of the local sub-graph of a node i that contains only the direct neighbors of node i, and is defined as [^2^](#_ENREF_2):

$$E_{loc,i}=\frac{\sum_{j,h\in N, j\neq i} a_{ij}a_{ih}\left[ d_{jh}(N_{i}) \right]^{-1}}{k_{i}(k_{i}-1)}$$

where d_jh_(N_i_) is the length of the shortest path between j and h which contains only neighbors of i.

The **Betweenness centrality** is the fraction of all shortest paths in the network that pass through a given node i and is defined as [^3^](#_ENREF_3):

$$b_{i}=\sum_{\begin{aligned} h,j\in N \\ h\neq j, h\neq i, j\neq i \end{aligned}} \frac{\rho_{ij}(i)}{\rho_{hj}}$$

where ρ_hj_ is the number of shortest paths between h and j, and ρ_hj_(i) is the number of shortest paths between h and j that pass through i.

The **Characteristic path length** is the average of all shortest paths between each possible pair in the network and defined as [^1^](#_ENREF_1):

$$L=\frac{1}{n}\sum_{i\in N} L_{i}=\frac{1}{n}\sum_{i\in N} \frac{\sum_{j\in N, j\neq i} d_{ij}}{n-1}$$

where d_ij_ is the shortest path length (distance) between nodes i and j, and L_i_ is the average distance between node i and all other nodes.

The **Global efficiency** is the average inverse shortest path length and defined as [^2^](#_ENREF_2):

$$E=\frac{1}{n}\sum_{i\in N} E_{i}=\frac{1}{n}\sum_{i\in N} \frac{\sum_{j\in N, j\neq i} d_{ij}^{-1}}{n-1}$$

where E_i_ is the efficiency of node i.

To estimate the property of network Small-worldness, a matched random network with same number of nodes and edges as the real network was generated.

The **Small-worldness** represents the network with a higher local clustering but nearly the same characteristic path length as random networks, and is defined as the ratio of normalized clustering coefficient (γ=C / C_rand_) and normalized characteristic path length (λ=L / L_rand_) [^1^](#_ENREF_1):

$$\sigma=\frac{C/{C_{rand}}}{L/{L_{rand}}}$$

where C and L are the average Clustering coefficient and the Characteristic path length of real networks, and C_rand_ and L_rand_ indicate the means of random networks. In general, the Small-world topological organization should meet the criteria of γ > 1 and λ ≈ 1, or σ = γ / λ > 1 [^4^](#_ENREF_4).

**References:**

1. Watts DJ, Strogatz SH. Collective dynamics of 'small-world' networks. *Nature*. 1998;393:440-442

2. Latora V, Marchiori M. Efficient behavior of small-world networks. *Physical review letters*. 2001;87:198701

3. Freeman LC. Centrality in social networks conceptual clarification. *Social Networks*. 1978;1:215-239

4. Humphries MD, Gurney K, Prescott TJ. The brainstem reticular formation is a small-world, not scale-free, network. *Proceedings. Biological sciences / The Royal Society*. 2006;273:503-511

5. Newman ME. Fast algorithm for detecting community structure in networks. *Physical review. E, Statistical, nonlinear, and soft matter physics*. 2004;69:066133

6. Newman ME. Assortative mixing in networks. *Physical review letters*. 2002;89:208701

**Supplementary Table 1** Carotid ultrasound characteristics of the vulnerable plaque patients and stable plaque patients

| Characteristics | vulnerable plaque (hypoechoic and isoechoic) | stable plaque (hyperechoic) |
| --- | --- | --- |
| Number of people(N) | 33 | 28 |
| Number of plaque/(N) | 52 | 42 |
| Main plaque side/ Left, (N) | 16 | 10 |
| CIMT | 0.80±0.10 | 0.82±0.10 |

**Supplementary Table 2.** Anatomical ROIs generated based on the AAL parcellation. Each of the 45 labels contains ROIs in both left and right hemispheres, which yield a total of 90 ROIs.

| Precentral gyrus | Lingual gyrus |
| --- | --- |
| Superior frontal gyrus (dorsal) | Superior occipital gyrus |
| Orbitofrontal cortex (superior) | Middle occipital gyrus |
| Superior frontal gyrus (medial) | Inferior occipital gyrus |
| Orbitofrontal cortex (medial) | Fusiform gyrus |
| Middle frontal gyrus | Postcentral gyrus |
| Orbitofrontal cortex (middle) | Superior parietal gyrus |
| Inferior frontal gyrus (opercula) | Inferior parietal lobule |
| Inferior frontal gyrus (triangular) | Supramarginal gyrus |
| Orbitofrontal cortex (inferior) | Angular gyrus |
| Rolandic operculum | Precuneus |
| Supplementary motor area | Paracentral lobule |
| Olfactory | Caudate |
| Rectus gyrus | Putamen |
| Insula | Pallidum |
| Anterior cingulate gyrus | Thalamus |
| Middle cingulate gyrus | Heschl gyrus |
| Posterior cingulate gyrus | Superior temporal gyrus |
| Hippocampus | Temporal pole (superior) |
| Parahippocampal gyrus | Middle temporal gyrus |
| Amygdala | Temporal pole (middle) |
| Calcarine cortex | Inferior temporal gyrus |
| Cuneus |  |
